# Reducing radiation dose to normal brain through a risk adapted dose reduction protocol for patients with favourable subtype anaplastic glioma

**DOI:** 10.1186/s13014-017-0782-3

**Published:** 2017-03-02

**Authors:** M. Back, M. LeMottee, C. Crasta, D. Bailey, H. Wheeler, L. Guo, T. Eade

**Affiliations:** 1 Northern Sydney Cancer Centre, Royal North Shore Hospital, Sydney, NSW Australia; 2 Central Coast Cancer Centre, Gosford Hospital, Gosford, NSW Australia; 3 Department of PET and Nuclear Medicine, Royal North Shore Hospital, Sydney, Australia; 4 Sydney Medical School, University of Sydney, Sydney, Australia; 5 Sydney Neuro-Oncology Group, Sydney, NSW Australia; 6 Department of Radiation Oncology, Northern Sydney Cancer Centre, Royal North Shore Hospital, St Leonards, Sydney, NSW 2065 Australia

**Keywords:** Anaplastic glioma, Dosimetry, Intensity modulated radiation therapy, Neurocognitive

## Abstract

**Aim:**

In patients with isocitrate dehydrogenase (IDH) mutated anaplastic glioma determine the dosimetric benefits of delivering radiation therapy using a PET guided integrated boost IMRT technique (ib-IMRT) compared with standard IMRT (s-IMRT) in reducing dose to normal brain.

**Methods:**

Ten patients with anaplastic glioma, identified as a favourable molecular subgroup through presence of IDH mutation, and managed with radiation therapy using an ib-IMRT were enrolled into a dosimetric study comparing two RT techniques: s-IMRT to 59.4Gy or ib-IMRT with 59.4/54Gy regions. Gross Tumour volume (GTV) and Clinical Target Volumes (CTV) were determined by MRI, 18F-Fluoroethyltyrosine (FET) and 18F-Fluorodeoxyglucose (FDG) PET imaging. A standard risk Planning Target Volume (PTVsr) receiving 59.4Gy (PTV59.4) in the s-IMRT technique was determined by MRI T2Flair and FET PET. For the ib-IMRT technique this PTVsr volume was treated to 54Gy, and the high-risk PTV (PTVhr) receiving 59.4Gy was determined as a higher risk region by FDG PET and MRI gadolinium enhancement. Standard dosimetric criteria and normal tissue constraints based on recent clinical trials were used in target delineation and planning. Normal Brain was defined as Brain minus CTV. Endpoints for dosimetric evaluation related to mean Brain dose (mBrainDose), brain volume receiving 40Gy (Brainv40) and 20Gy (Brainv20). The variation between the dosimetric endpoints for both techniques was examined using Wilcoxon analysis.

**Results:**

The 10 patients had tumours located in temporal (1), parietal (3), occipital (2) and bifrontal (4) regions. In ib-IMRT technique the median volume of PTVhr was 25.5 cm3 compared with PTVsr of 300.0 cm3. For dose to PTVhr the two treatments were equivalent (*p* = 0.33), and although the ibIMRT had a prescribed 10% dose reduction from 59.4Gy to 54Gy the median reduction was only 5.9%. The ib-IMRT dosimetry was significantly improved in normal brain endpoints specifically mBrainDose (*p* = 0.007), Brainv40 (*p* = 0.005) and Brainv20 (*p* = 0.001), with a median reduction of 9.3%, 19.0 and 10.8% respectively. After a median follow-up of 38 months two patients have progressed, with no isolated relapse in the dose reduction region.

**Conclusion:**

An approach using ib-IMRT for anaplastic glioma produces significant dosimetric advantages in relation to normal brain dose compared with s-IMRT plan. This is achieved without a significant reduction to the target volume dose despite the reduction in prescribed dose. This technique has advantages to minimise potential late neurocognitive effects from high dose radiation in patients with favorable subtype anaplastic glioma with predicted median survival beyond ten years.

## Introduction

Anaplastic oligodendroglial tumours are a favourable subsite of high grade glioma that have recently been shown to be associated with median survival beyond 10–15 years with management by combined modality therapy with limited surgery, radiation therapy and chemotherapy [1, 2]. In recent years these and other patients whose tumours are harbouring an isocitrate dehydrogenase (IDH) mutation are being classified into a more favourable subgroups of patients with prolonged survival [3–5]. Patients are often diagnosed at a younger age and present with symptoms such as limited seizure activity without major morbidity or affect on performance status. The more durable survival in this high functioning younger population places a greater emphasis on optimizing therapy with a balance between tumour control and minimization of late morbidity. Although favourable prognosis with variable growth rate, the tumour is often quite extensive at initial presentation with MRI demonstrating widespread T2 Flair change surrounding the initial T1 dense or enhancing mass [6, 7]. Radiation therapy is the key component of management but high dose therapy and large volume standard treatment has been associated with late neurocognitive effects that will reach clinical significance in patients with durable survival [8, 9].

Technological improvements in diagnostic imaging, nuclear medicine and radiation therapy treatment can potentially deliver a more targeted dose of radiation to the tumour and spare normal brain [10]. Similarly in favourable anaplastic glioma (AG) there may be a less aggressive region of the tumour that may be managed with lower radiation dose as utilized in primary low grade glioma.

This study aims to evaluate the dosimetric benefits of a novel radiation approach for favourable AG utilizing improved targeting of radiation therapy (intensity modulated radiation therapy) to areas within the tumour at different dose levels (integrated boost) defined by MRI and nuclear medicine techniques (dose painting).

## Methods

Consecutive adult patients diagnosed with AG and referred to The Department of Radiation Oncology at the Northern Sydney Cancer Centre were entered into a prospective database, approved by Institutional Ethics Review Board. 10 patients with favourable molecular subgroup as defined by presence of IDH mutation were included in this dosimetric study. These patients were formally managed with definitive radiation therapy under the departmental IMRT Integrated Boost Protocol (ib-IMRT) adapting dosimetric criteria and constraints specified as per the standard EORTC CATNON Protocol [11]. For these 10 patients an additional plan was produced using a standard IMRT (s-IMRT) plan without integrated boost; and these plans were then utilised for formal comparison.

### Patient selection

Eligible patients for the ib-IMRT Protocol were newly diagnosed or RT naive relapsed patients diagnosed with an anaplastic glioma that had favourable prognosis as defined by molecular features of IDH1 mutation or 1p19q co-deletion.

### Pretreatment preparation

Patients required a gadolinium enhanced 3T MRI in the six weeks prior to commencement of radiation therapy. Axial T1, T2Flair and T1gad sequences were utilized for RT Planning.

Nuclear medicine imaging with combined FET and FDG PET were performed. The FET studies were acquired on a Siemens Biograph mCT PET/CT scanner with extended axial FoV and time of flight (ToF) imaging capability, with 20-min dynamic and 10-min static acquisitions following a 3 min FET infusion period. The standard injected dose of FET was 150 MBq.

Reconstruction parameters included 3D OSEM with two iterations and 21 subsets with PSF recovery and ToF. Scatter correction was model-based and attenuation correction was CT-based. Post-reconstruction Gaussian filtering was performed with filter size of 5 mm. The FDG PET involved both immediate and 4 h delayed sequences.

As based on the European guidelines, the FET-PET was reported as pathological if the ratio of uptake was 1.6 times the background uptake of healthy brain tissue [12].

### Radiation therapy planning

The patients had CT simulation with immobilization by an individual Kevlar thermoplastic mask system. MRI and PET scans were fused with the non-contrast CT scan and entered into the Varian Eclipse Planning system. Any historical MRI imaging, especially for patients with progressive disease after prior surveillance was also imported for assistance with target volume determination. The quality of fusion was confirmed with comparison of manually generated structures (surgical cavity, ventricular system and pons) using the non-contrast planning CT as the reference. A single clinician and dosimetrist were used for the planning process.

### Target volume segmentation for ib-IMRT

The gross tumour volume was developed from a combination of volumes generated from each of the imported MRI sequences; specifically Gross Tumour Volume (GTV) T1, GTVT2F, GTVT1gd, GTVFET and GTVFDG. The GTVT2F included all of the T2Flair evident except any underlying presumed chronic periventricular changes.

The GTVFET was generated manually based on uptake normalized to the blood flow uptake in the sagittal sinus. The GTVT2F was correlated with the GTVFET to clarify areas of subtle T2Flair or alternatively subtle FET uptake.

Subsequently high and standard risk GTVs were developed for the integrated boost and named GTVhr and GTVsr as corresponding to the dose levels 59.4Gy and 54Gy. The higher risk region GTVhr comprised the GTVT1gd (any pre or postop enhancing tumour) and GTVFDG. The standard risk region GTVsr comprised the GTVhr as well as GTVT2F and GTVFET. The GTVsr was then reviewed with GTVT1 and the surgical cavity on planning CT to confirm coverage; though the surgical cavity was excluded if there was a large CSF collection contiguous with the dura.

If the T2Flair change was noted to have FET uptake, then it was managed as non-enhancing tumour and included in the GTVsr, receiving a prescribed dose of 54Gy. If no FET uptake was evident (and no FDG uptake or increased T1density) then the T2 Flair was presumed to be edema or gliosis and was not routinely incorporated into the GTVsr. As the MRI was the key diagnostic modality to define target volumes then if there was FET uptake, but at a lower volume, within the T2 Flair then the whole of the MRI abnormality was included within the GTVsr. However if there was FET uptake outside of the T2Flair, the protocol would include that region in the GTVsr.

A high-risk clinical target volume (CTVhr) was created from GTVhr and expansion of 2 mm. The standard risk clinical target volume (CTVsr) was created from GTVsr and expansion of 10 mm. Both were restricted at anatomical boundaries.

Final planning target volumes (PTVhr and PTVsr) were created from corresponding CTV with an additional expansion of 0 mm and 3 mm respectively.

There is specifically no expansion to the PTVhr as it is understood that the CTVhr is always covered by the CTVsr and this latter volume is expanded by 3 mm. Thus any setup error within the 3 mm measured departmental error for IGRT will mean the PTVhr will be in the region receiving 54–59.4Gy.

### Normal tissue segmentation

Multiple normal tissue structures were generated based on the T1 MRI but reviewed with the planning CT and modified if variation. IMRT Planning priorities were weighted to dose tolerance placed on optic chiasm and nerves (maximum 5500 cGy) and brainstem (5500 cGy with <1 cm3 allowed at 6000 cGy). For the PTV structures, 100% of dose had to be delivered to 95% of the target volume. Additionally hippocampal structures were delineated to encompass the surrounding medial temporal lobe. The dose to the contralateral hippocampus was minimized to a mean dose range between of 15Gy-20Gy. The ipsilateral hippocampus was restricted to this dose level if uninvolved by PTVsr; and optimally a mean dose of 40Gy if partially involved.

### ib-IMRT Plan generation

The dose prescription for the ib-IMRT plan was 59.4Gy and 54Gy in 33 fractions delivered to PTVhr and PTVsr over six and a half weeks. Coverage of 95% of the specific PTV by 100% of the dose was the dosimetry target.

An integrated boost IMRT plan was created with inverse planning limited by a maximum of 6 fields and dose constraints with highest priority on PTVhr, optic chiasm and brainstem. At sites where PTVhr involved a dose limiting structure, a separate high priority PTV was created for the overlap region and optimisations performed to control the dose at that region. Fluence painting was undertaken on each field to remove areas of high dose gradient. This plan was used for treatment delivery.

### Radiation therapy delivery

Treatment was delivered with IMRT using 6MV photons on a Varian Trilogy or TrueBeam STx Linear Accelerator. Daily IGRT was performed with the on-board imager (OBI) verifying position based on middle cranial fossa and orbital bone landmarks, and weekly Cone Beam CT to exclude any significant shift of intracranial contents arising from oedema, intercurrent haemorrhage or tumour reduction.

### Systemic therapy management

Patients were offered adjuvant temozolomide (TMZ) for six months following the completion of RT. No concurrent chemotherapy was used with RT.

### Study comparison plans

For the clinical study comparison plans were produced for s-IMRT using an inverse planned IMRT technique based on the generated PTVsr without a dose reduction. For these plans the PTVsr was assigned a dose of 59.4Gy in 33 fractions so that all known tumour would be covered by the dose used in the EORTC CATNON Protocol [11].

### Dosimetric endpoints

The primary dosimetric endpoint for the study is the mean Brain dose (defined as Whole Brain minus PTVsr). Secondary Brain Dose parameters include percentage volume of brain receiving 40Gy (BrainV40) and volume of Brain receiving 20Gy (BrainV20).

Other dosimetric secondary endpoints relate to dose received by the high risk region (GTVhr) and contralateral hippocampus for both sets of plans.

### Clinical endpoint

All patients were managed with the ib-IMRT plan. Any treatment failure within the PTVhr or PTVsr was recorded, especially in regards to isolated relapse within the region of dose reduction to 54Gy (PTVsr).

### Statistical considerations

All patients had clinical and dosimetric data entered on an Excel database at Northern Sydney Cancer Centre and updated for outcome events.

The variation in specific dosimetric endpoints between the ib-IMRT and s-IMRT dosimetric endpoints was examined using Wilcoxon analysis. Significance was determined at the *p* = 0.05 level.

## Results

The ten patients all received the prescribed radiation dose with the ib-IMRT plan without any interruption or dose modification.

The individual patient clinical and tumour characteristics are detailed in Table 1. The tumours were distributed in the brain with locations in frontal (4), parietal (3), temporal (1), occipital (2) lobes. There was evidence of gad enhancement on MRI in 4 patients, and FDG uptake in 7 patients. There was evidence of FET uptake in all ten patients that was confirmed as correlating with the T2Flair on MRI.

**Table 1:** Patient Clinical Characteristics

	Subgroup	Number
Age at Diagnosis	<40 year	2
	>40 year	8
	Median	46 years (35–62 years)
Tumour Site	Frontal	4
	Parietal	3
	Temporal	1
	Occipital	2
Pathology	AOD	4
	AOA	6
FDG Uptake	Nil	3
	Positive	7
Gad enhancement	Nil	6
	Positive	4
Tumour Volume High Risk (PTVhr)	Median Range	25.5 cm3 (7.0–142.1 cm3)
Tumour Volume Standard Risk (PTVsr)	Median Range	300.0 cm3 (119.2–618.1 cm3)
Tumour Volume Ratio of High to Low Risk (PTVhr/PTVsr)	Median Range	11.5% (3.2–46.4%)

The diffuse extent of the tumours was evident with the volume of PTVsr which ranged from 119 cm3 to 618 cm3 with a median of 300 cm3.

### Radiation planning

All generated plans achieved the minimum dosimetric criteria for PTVsr coverage and normal tissue tolerances in brainstem, optic chiasm and contralateral hippocampus. The doses received by the high-risk tumour region (PTVhr) were equivalent in the two planning techniques (*p* = 0.33). As expected the dose reduction to the PTVsr caused a significant reduction of dose in the integrated boost technique (*p* < 0.001); however the 10% prescribed dose reduction resulted in only a median 5.9% reduction to the mean dose received by this region.

### Dosimetric endpoints

The dosimetric brain endpoints were significantly improved for all normal brain dose categories with the ib-IMRT plans compared with the s-IMRT plans. The results are summarized in Tables 2 and 3.

**Table 2:** Dosimetric endpoints for ibIMRT and sIMRT Plans

Endpoint	ibIMRT (median and range)	sIMRT (median and range)	*P* value
PTVhr Dose (Median)	59.9Gy (58.0–60.4Gy)	59.8Gy (58.1–61.6Gy)	*p* = 0.33
PTVsr Dose (Median)	56.0Gy (54.1–57.0Gy)	59.8Gy (58.1–61.6Gy)	*P* < 0.001
V40 (volume of brain receiving 40Gy)	14.9% (11.8–30.4%)	18.3% (14.5–29.2%)	*p* = 0.005
V20 (volume of brain receiving 20Gy)	45.8% (29.2–71.1%)	52.6% (35.2–68.4%)	*p* = 0.001
Mean Dose to Brain	22.3Gy (16.3–29.9)	24.5Gy (19.3–30.9)	*p* = 0.007

**Table 3:** Percentage Change between ibIMRT and sIMRT Plans for each patient

Endpoint	Median% difference between ibIMRT and sIMRT Plans	Range of results
Mean Brain Dose Change	9.3%	1.0–17.8%
Mean V40 Change	19.0%	5.5–40.2%
Mean V20 Change	10.8%	1.0–25.8%
Mean Contralateral Hippocampal Dose Change	13.5%	−3.5–41.0%
Mean PTVsr dose reduction in ibIMRT Plan	5.9%	3.9–9.3%

**Fig. 1:**
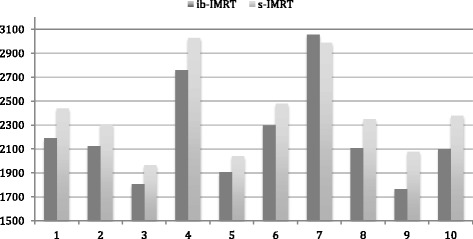
Mean Brain Dose (cGy): Comparison of ibIMRT and sIMRT Plans for each patient

**Fig. 2:**
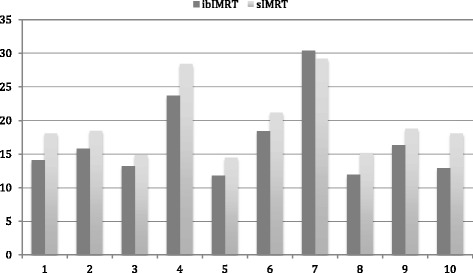
V40 Brain Levels (%): Comparison of ibIMRT and sIMRT Plans for each patient

The reduction in mean brain dose for each of the patients is shown in Fig. 1, with a median reduction of 9.3% (range 1.0 to 17.8%). The impact of the integrated boost planning technique was demonstrated at the BrainV40 levels with the reduction of the volume of brain receiving 40Gy being significantly decreased by a median level of 19.0%, and with reductions up to 40.2% (Fig. 2). The lower dose to the normal brain, as demonstrated by the BrainV20 levels, paralleled the reduction of the higher dose levels with a median reduction of 10.8% (range 1.0 to 25.8%). Similarly the contralateral brain hippocampal doses were calculated, and did show a reduction in mean dose with the integrated boost technique that parallels the V20 Brain dosimetric improvements (Table 3).

**Fig. 3:**
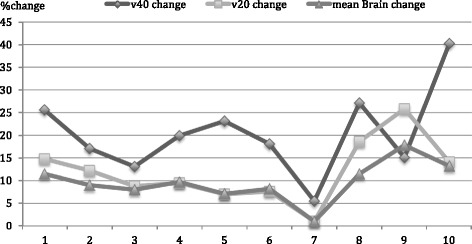
Percentage Change of normal brain endpoints between ibIMRT and sIMRT Plans for each patient

Only one integrated boost plan had no impact on dosimetric endpoints of normal brain dose (Patient 7), and that was the patient with the largest PTVsr and highest normal brain doses (Fig. 3).

Specifically when averaged across the study cohort, a prescribed reduction of dose by 5.4Gy or 10% to the low risk region (PTVst) in the ib-IMRT plan produced a proportional reduction of only 5.9% to the actual dose in that region. However this dose reduction planning technique resulted in a much higher proportional reduction of dose to normal brain by 9.3% (for mean brain dose) and a reduction in the volume of brain being treated to 40Gy by 19.0%.

### Clinical endpoint

At a median follow-up of 38 months in this patient cohort, two patients have had tumour progression. One was distant failure outside of both PTVsr; and the other had progression within both PTVhr and PTVsr.

## Discussion

This dosimetric study confirms the benefits of utilizing an integrated boost approach to IMRT as an attempt to minimise the amount of radiation therapy delivered to normal brain tissue in patients with anaplastic glioma. The volume of normal brain receiving doses above 40Gy was reduced by a median of 19%, with significant reductions to the mean brain dose with this technique compared to standard IMRT. These dosimetric improvements have the potential to minimise the risk of late neurocognitive effects from RT by impacting on the predictive factors of radiation dose, dose per fraction and volume of normal brain irradiated.

Although the relationship of dose tolerance and pathophysiology of cortical brain tissue radiation effect is poorly defined in adults, the dose level of 40Gy delivered over 6 weeks is significant as a moderate dose to normal brain that may give rise to either impaired neuronal differentiation or cerebrovascular impairment [13]. In patients managed with RT in the 1990s for low grade glioma there was an association with higher dose per fraction and treated volume for late neurocognitive deficits [9]. More recently volumetric analysis of cerebral cortex following fractionated partial brain RT showed a dose dependent atrophy of cortex with doses beyond 35Gy [14]. RT techniques have improved in recent years with superior target delineation based on digital technology and MRI imaging that may impact on historical volume of brain tissue irradiated [15]. However the dose delivered in anaplastic glioma can less be modified given presumed dose response models for high-grade glioma, unless there a risk-adapted approach can be implemented as per this current study. Reduction of dose at this level by utilizing integrated boost planning, without impacting negatively on dose to tumour or low dose to normal brain, can thus provide the potential for less morbidity.

For patients diagnosed with IDH mutated anaplastic glioma the potential for survival extending beyond 10–15 years is high based on the long term outcome of the RTOG9402 and EORTC26951trials published in 2013 [1, 2]. Despite these two studies being commenced in the late 1990s before the improvements in digital imaging and radiation dosimetry the use of adjuvant RT and chemotherapy resulted in median survivals of 14.7 years and beyond 11.7 years respectively for patients with codeleted tumours. Future protocols need to consider techniques that minimise late morbidity, as there is now a population of patients who will be alive to experience the late impact of large volume radiation.

Another issue related to these tumours long natural history is the extensive nature of disease at initial presentation with non-enhancing tumour extending beyond the region that has transformed to high grade disease or more densely clustered cells. Tumour extend across the corpus callosum is not infrequent and may not only give rise to neurocognitive deficits at presentation but also because to the large volumes needed for irradiation place a higher risk of late treatment morbidity. It is estimated that the majority of patients with primary brain tumour will have deficits on neuropsychological testing, the aetiology not limited to neuronal damage from tumour or treatment but also other factors such as seizure activity, anticonvulsants and neuro endocrine effects [7, 8, 16, 17].

The current protocol delivered a dose of 54Gy for the low risk region as defined by absence of Gadolinium or FDG uptake. The radiation dose response for anaplastic glioma is poorly defined with doses historically correlating with glioblastoma multiforme. However for low grade glioma studies reviewing dose response have shown no advantage for 64Gy versus 50.4 Gy [18] or 45 Gy versus 54Gy [19]. Thus the use of 54Gy in the area of glioma that has imaging features consistent with low grade tumour can be justified.

MRI imaging remains the foundation for RT Planning of brain tumours, though there are limitations in MRI diagnosis as it is only partially able to determine the metabolic activity of tumours. Generally blood brain barrier (BBB) disruption and subsequent contrast enhancement on MRI T1 sequences indicates the extent of high-grade tumour. However in the scenario of non-enhancing tumour where the BBB remains intact the extent of disease may be difficult to clarify [20].

Improving the therapeutic ratio of tumour control to normal tissue morbidity through approaches such as this integrated boost protocol is dependent upon accurate target volume delineation. Differentiating the T2 Flair change from perilesional edema or non-enhancing tumour is one of the difficult aspects of radiation treatment design for patients with IDH mutated anaplastic gliomas; and the proposed role of the FET PET is to determine the nature of the T2 Flair component. It is believed that FET uptake will reflect that this is likely to be predominantly tumour and less likely to be edema alone. Thus in the described protocol the T2Flair is then treated to 54Gy. For radiation oncologists whose protocols do not routinely include perilesional edema, or those who dose reduce to 46Gy the perilesional edema, then it is clinically important to be able to define this MRI change.

In the protocol, the higher dose region for the integrated boost was designed to optimize radiation dose to the areas of higher biological activity. Whilst amino-acid tracers have specificity in determining geographical extent of tumour in normal brain, the scan may be less reliable in indicating a region of higher metabolic activity or tumour grade. In the absence of established data or more efficacious tracers, the higher glucose metabolism in FDG PET was used to determine regions of higher grade (in addition to any MRI gadolinium enhancement) for the integrated boost. This is similar to the role of FDG PET to determine increased tumour grade in patients being managed following radiological diagnosis of a low grade glioma.

Future opportunities exist in exploring novel PET tracers that may isolate further regions in the tumour that are of high risk and be targeted for dose painting even beyond the standard 59.4Gy level. These may be hypoxia tracers or improved amino acid tracers to identify areas of higher proliferation [21]. Also with the majority of these favourable glioma expressing an IDH1 mutation, tracers targeting this protein may provide more specific determination of target volume to potentially reduce extent of CTV.

## Conclusions

An approach using ib-IMRT for anaplastic glioma produces significant dosimetric advantages in relation to normal brain dose compared with s-IMRT. This is achieved without a significant reduction to the target volume dose despite the reduction in prescribed dose. This technique has advantages to minimise potential late neurocognitive effects from high dose radiation in patients with favorable subtype anaplastic glioma with predicted median survival beyond ten years.

## References

[1] Cairncross G, Wang M, Shaw E (2013). Phase III trial of chemoradiotherapy for anaplastic oligodendroglioma: long-term results of RTOG 9402. J Clin Oncol.

[2] van den Bent MJ, Brandes AA, Taphoorn MJ (2013). Adjuvant procarbazine, lomustine, and vincristine chemotherapy in newly diagnosed anaplastic oligodendroglioma: long-term follow-up of EORTC brain tumor group study 26951. J Clin Oncol.

[3] Yan H, Parsons DW, Jin G (2009). IDH1 and IDH2 mutations in gliomas. N Engl J Med.

[4] Cairncross JG, Wang M, Jenkins RB (2014). Benefit from procarbazine, lomustine, and vincristine in oligodendroglial tumors is associated with mutation of IDH. J Clin Oncol.

[5] Olar A, Wani KM, Alfaro-Munoz KD (2015). IDH mutation status and role of WHO grade and mitotic index in overall survival in grade II-III diffuse gliomas. Acta Neuropathol.

[6] Eckel-Passow JE, Lachance DH, Molinaro AM (2015). Glioma groups based on 1p/19q, IDH, and TERT promoter mutations in tumors. N Engl J Med.

[7] Hathout L, Pope WB, Lai A (2015). Radial expansion rates and tumor growth kinetics predict malignant transformation in contrast-enhancing low-grade diffuse astrocytoma. CNS Oncol.

[8] Taphoorn MJ, Schiphorst AK, Snoek FJ (1994). Cognitive functions and quality of life in patients with low-grade gliomas: the impact of radiotherapy. Ann Neurol.

[9] Klein M, Heimans JJ, Aaronson NK (2002). Effect of radiotherapy and other treatment-related factors on mid-term to long-term cognitive sequelae in low-grade gliomas: a comparative study. Lancet.

[10] Vigliani MC, Sichez N, Poisson M, Delattre JY (1996). A prospective study of cognitive functions following conventional radiotherapy for supratentorial gliomas in young adults: 4-year results. Int J Radiat Oncol Biol Phys.

[11] Van Den Bent MJ, Erridge S, Vogelbaum M et al. Results of the interim analysis of the EORTC randomized phase III CATNON trial on concurrent and adjuvant temozolomide in anaplastic glioma without 1p/19q co-deletion: An Intergroup trial. J Clin Oncol 34, 2016 (suppl; abstr LBA2000)

[12] Vander Borght T, Asenbaum S, Bartenstein P (2006). EANM procedure guidelines for brain tumour imaging using labelled amino acid analogues. Eur J Nucl Med Mol Imaging.

[13] Douw L, Klein M, Fagel SS (2009). Cognitive and radiological effects of radiotherapy in patients with low-grade glioma: long-term follow-up. Lancet Neurol.

[14] Karunamuni R, Bartsch H, White NS (2016). Dose-dependent cortical thinning after partial brain irradiation in high-grade glioma. Int J Radiat Oncol Biol Phys.

[15] Corn BW, Yousem DM, Scott CB (1994). White matter changes are correlated significantly with radiation dose. Observations from a randomized dose-escalation trial for malignant glioma (Radiation Therapy Oncology Group 83–02). Cancer.

[16] McAleer MF, Brown PD (2015). Neurocognitive function following therapy for low-grade gliomas. Semin Radiat Oncol.

[17] Aaronson NK, Taphoorn MJ, Heimans JJ (2011). Compromised health-related quality of life in patients with low-grade glioma. J Clin Oncol.

[18] Shaw E, Arusell R, Scheithauer B (2002). Prospective randomized trial of low- versus high-dose radiation therapy in adults with supratentorial low-grade glioma: initial report of a North Central Cancer Treatment Group/ Radiation Therapy Oncology Group/Eastern Cooperative Oncology Group study. J Clin Oncol.

[19] Karim AB, Maat B, Hatlevoll R (1996). A randomized trial on dose–response in radiation therapy of low-grade cerebral glioma: European Organization for Research and Treatment of Cancer (EORTC) Study 22844. Int J Radiat Oncol Biol Phys.

[20] Götz I, Grosu AL (2013). 18F FET-PET imaging for treatment and response monitoring of radiation therapy in malignant glioma patients - a review. Front Oncol.

[21] Grosu AL, Weber WA (2010). PET for radiation treatment planning of brain tumours. Radiother Oncol.

